# The Dreamland: Validation of a Structured Dream Diary

**DOI:** 10.3389/fpsyg.2020.585702

**Published:** 2020-10-16

**Authors:** Brigitte Holzinger, Lucille Mayer, Isabel Barros, Franziska Nierwetberg, Gerhard Klösch

**Affiliations:** ^1^Institute for Consciousness and Dream Research, Vienna, Austria; ^2^Psychology Department, California State University, Long Beach, CA, United States; ^3^Department of Neurology, Medical University of Vienna, Vienna, Austria

**Keywords:** dream, DL-Q, Hall and van de Castle Coding System, sleep laboratory, research instrument development, questionnaire, dream diary

## Abstract

Validated instruments for the analysis of dream contents are still scarce. Therefore, the aim of this study was to validate the Dreamland Questionnaire (DL-Q) by comparing its results to those of the Hall and van de Castle Coding System (HVDC). Twenty-two participants voluntarily filled in a written dream report as well as our DL-Q questionnaire, in total 30 dreams were collected with both measures. Written reports were analyzed with the HVDC and results of the two instruments were compared using Pearson correlations. Results showed that correlations were high for dominant characters, pleasantness of dream content, and body-related experiences. However, some DL-Q items showed low correlations and others could not be compared directly, as the HVDC did not include the same set of items. The DL-Q showed satisfactory validity and reliability as a measure of dream criteria and may serve as an effective tool for diagnosis and evaluation and facilitate future clinical and research studies. Nevertheless, some items could not be compared as part of this study and should be validated in future investigations.

## Introduction

A variety of methodologies has been used for the collection and analysis of dream reports, each covering different aspects of dreaming (Klösch and Holzinger, 2014). Dream reports differ as a result of setting, awakening method, collection technique, and analysis. Private settings produce markedly distinct reports from laboratory setting and influence dream recall (Foulkes, 1996; Schredl et al., 2003). Awakenings can be forced or spontaneous, and forced awakenings differ in the stimulus with which the dreamer is awakened (Dement and Wolpert, 1958; Klösch and Holzinger, 2014). Dream reports can be collected on audio tape, in more detailed written form (e.g., dream diary), in the more structured form of questionnaires, or even in the form of drawings and enactments (Schredl, 2002; Klösch and Holzinger, 2014). Using the therapeutic setting to discover the underlying meaning of a dream is an additional possibility to discover one’s dream world. Furthermore, dreams can be collected on a daily basis or in retrospect (Schredl, 1999; Watson, 2003; Zadra and Geneviève, 2012). Several methodological shortcomings (e.g., how to interpret pictures objectively) of one or the other method often led to a combination of different techniques. However, scientists mostly rely on questionnaires and written reports, with dream diaries being considered as the most important source of information regarding dream characteristics (Klösch and Holzinger, 2014). The recalled dream content is usually first recorded on tape and written down later or written down directly in a dream diary (e.g., Foulkes, 1979; Hurovitz et al., 1999). Although most scientists in the field of dream research use dream diaries as a major instrument for the collection and analysis of dreams, basic literature on how to use and organize dream diaries is still scarce (Strauch and Meier, 1992; Schredl, 1999). Another major limitation of written dream reports is the lack of standardized procedures to collect and analyze written protocols (e.g., Smith, 1984).

One coding system that allows for a comparison of dreams is the Hall and Van de Castle Coding System (HVDC), which was developed in 1966 (Hall and Van de Castle, 1966). Overall, the HVDC is one approach with solid empirical support. However, the system has its weaknesses: short (less than 50 words) and long dreams (more than 300 words) cannot be analyzed. Learning how to use the coding system, i.e., getting to know all available categories, and the analysis of big samples is a quite time-consuming task.

To allow for a less demanding, more structured analysis of dreams, a large number of dream questionnaires has been developed. Unfortunately, most of them are still lacking standardization and validation (e.g., Hauri et al., 1967; Kallmeyer and Chang, 1997; Schredl, 1999, Schredl, 2004). Many questionnaires focus on specific topics such as dream motifs (e.g., Yu, 2012; Malinowski and Horton, 2014), nightmares (e.g., Belicki, 1992), impactful events and traumas (e.g., Orsillo et al., 2007), emotionality (e.g., Rezzonico and Liccione, 2004; Zadra et al., 2006; Yu, 2007), lucid dreaming (e.g., Voss et al., 2013), REM sleep behavior disorders (e.g., Boeve et al., 2011), or assess dreaming in general but in a rather complex manner (e.g., Kallmeyer and Chang, 1997; Aumann et al., 2012). One reason for the lack of validation is that several questionnaires refer to personal constructs or traits which are difficult to evaluate with other instruments (e.g., Hartmann’s concept of thick and thin boundaries, Hartmann et al., 1998). Nevertheless, in dream research there is a need of an easy-to-use instrument, which can be completed by patients as well as healthy subjects over longer time-periods. It should provide basic information regarding the formal criteria of the dream (frequency, length, time, etc.) as well as the content of the dream (themes, sources, emotional impact, the dreamer’s involvement in the dream etc.).

For this purpose, we aimed to validate the Dreamland (DL-Q), a 14-item self-report dream questionnaire that enables to investigate the subjective dream experience in retrospect (see Supplementary Appendix). Although it is structured like a questionnaire, it also possesses the functions of a dream diary. It is a comprehensive, easy-to-use tool that can be used as a screening or monitoring instrument in a therapeutic setting, in the field of consciousness, sleep and dream research, or as a complementary tool for the diagnosis of sleep disorders and other psychological impairments. The original version of the questionnaire was developed as early as 1997, now more than 20 years ago, and derived from our clinical observations, previous empirical work, and existing questionnaires. Its items were selected based on expert ratings on which dream aspects are the most important and central. Dreamers were asked which items were missing for an adequate and comprehensive description of their individual dreaming experience. The goal was to create an instrument that, one the one hand, comprises all relevant dream aspects, but on the other hand, is as short and quick to fill out and evaluate as possible. Therefore, some items that are included in other questionnaires or in the HVDC, are not part of the DL-Q. As a result, the application, evaluation and interpretation are significantly faster than for written dream reports and no specific training is necessary. Since its first use, the DL-Q has been developed further: some items were rephrased, some of the response categories were replaced with visual analog scales (VAS), and the questionnaire was improved with the help of test theorists. Fourteen items assess the dream characteristics and additionally, the back side of the questionnaire can be used for a more detailed description of the dream. This allows a direct comparison of dreamers’ ratings of their own dream and the ratings of experts with the help of a coding system, e.g., HVDC. In this sense, the questionnaire reflects our understanding of a multidimensional approach in dream research. Since its first development in 1997, the DL-Q has proven its usefulness in studies with patients as well as healthy subjects and in the comparison of different subpopulations (Klösch et al., 1999a,b; Holzinger et al., 2001, 2015; Lorenzo et al., 2002). We suggest that the DL-Q might be able to provide additional information about the nature of certain psychological disorders, and found, for instance, that the emotionality and thought content typical for eating disorders such as aggression in anorexics are also experienced in dreams (Holzinger et al., 2015). The DL-Q has been used in different therapeutic settings and most patients confirmed that they preferred to fill out the questionnaire over a written dream report. The shortness and structure of the DL-Q may help certain subpopulations struggling with cognitive impairments such as short attention span and contains clear item responses. The DL-Q was also shown by other researchers to discriminate successfully between dream recall and content under the influence of different drugs and without medication (Lorenzo et al., 2002). The questionnaire can easily be integrated into a sleep diary or combined with objective sleep measurements such as polysomnography or ambulant activity monitoring by actigraphs (Klösch et al., 2001). The DL-Q constitutes the basic item collection to analyze dream content, but items can be added easily in order to investigate more specific research questions.

## Methods

### Procedure and Sample

The aim of this study was to compare the results of the DL-Q to those obtained by the HVDC coding system. In order to analyze the written dream reports with the HVDC, the completion of a specific training is required. Therefore, we collaborated with specialists namely Bill Domhoff, Adam Schneider and IB, all working in the United States. To make this possible, the questionnaire was translated to English and all study participants were native English speakers. All the items included in both methods were selected and correlations were calculated.

In total, 22 participants took part in the study (12 women) aged between 24 and 65 (*M*_age_ = 34.32, SD_age_ = 11.61). Some provided more than one dream, which resulted in a collection of 30 dreams. Participants were recruited via E-mail and personal contacts, participation was voluntary. Before filling in the questionnaires, they were informed about the purpose of the study. All dreamers completed the questionnaire in the time frame from 21.09.17 – 16.05.18. Participants did not report any sleep disorders, psychological or physiological disorders, nor psychiatric history or substance abuse. Participants were informed that the study results would be used in research and published at a later date. They were instructed to fill in the questionnaire within 30 min after waking up. Participants were not educated about dreams in any way and we did not differentiate beforehand between levels of complexity and fragmentation. Since participation was completely anonymous and no names or other information was given, no ethical committee was consulted.

### Measures

#### The Hall and Van de Castle Coding System

The HVDC system consists of ten general categories, many of which are divided into subcategories: (1) Characters; (2) Social Interactions; (3) Activities; (4) Striving: Success and Failure; (5) Misfortune and Good Fortunes; (6) Emotions; (7) Physical Surroundings, Settings and Objects; (8) Descriptive Elements; (9) Food and Eating; (10) Elements of the Past. Interrater reliability between scores has been found to vary between categories, with the lowest percentage of perfect agreement for the scale “Social Interactions” (54–64%). For the analysis, the MS-Excel^TM^ – spreadsheet developed by Schneider and Domhoff (1995) can be used and allows for calculation of frequencies and certain indexes. After the coding procedure, individual dreams can be compared with those of a normative population (e.g., age- and sex-matched controls).

#### Dreamland Questionnaire (DL-Q)

The DL-Q comprises 14 items, pooled into three parts: In the first part, seven questions help assessing the number, duration, time of occurrence, perception, recall of dreams and awakenings due to dreams. In the second part, subjects are asked to write down last night’s most prominent dream and to characterize it by means of a set of given categories related to the dream content, degree of participation, affectivity as well as sensory and emotional involvement. Finally, the last part contains two items regarding lucid dreaming (Klösch and Holzinger, 2014). More detailed information and response categories can be found in the Supplementary Appendix. Only items 4, 8, and 9 allow multiple answers. Items 10 and 11 consist of visual analog scales (VAS). Results are analyzed by transferring the marked questions into a MS-Excel^TM^ – spreadsheet.

### Statistical Analyses

To see whether the correlation between corresponding items of the DL-Q and HDVC was sufficient, point-biserial correlations were calculated. All statistical analyses were carried out with the Statistical Package for Social Science 26 (SPSS, IBM Corp, 2017). On SPSS, point-biserial correlations were calculated as Pearson correlations. For statistical analysis, the threshold for the rejection of the null hypothesis was set to 0.05.

## Results

This analysis only included those items of the DL-Q with a counterpart in the HVDC. Therefore, only the second part of the DL-Q could be included, in which one dream of last night was described in more detail by the participants. Pearson correlations were high for some items, results are shown in Table 1. The appearance of animals, friends, family, unknown towns showed significant overlap in both measures. Body-related sensory impressions were correlated significantly with the physical and motor activity captured by the HVDC. The rating of the dream-plot as pleasant or unpleasant was also correlated in both instruments. The remaining items did not show significant concordance or could not be included in the analysis, since there were no corresponding items when comparing the DL-Q with the HVDC.

**TABLE 1:** Pearson correlations for corresponding items of DL-Q and HVDC.

Item DL-Q		Item HVDC	Pearson correlation *r*
What appeared in your dream?	Animals	Character: animal	0.937**
	Friends	Character: friends	0.667**
	Family	Character: family	0.696**
	Friends + colleagues + acquaintances + family	Character: familiar	0.196
	your own home + familiar building + known towns/villages + known objects	Setting: familiar	0.226
	Unknown towns/villages + indeterminable location + unknown objects	Setting: unfamiliar	0.388*
Please try to characterize the predominant types of sensory impressions:	Visual	Activity: S (visual)	–0.183
	Verbal	Activity: V (verbal)	–0.323
	Music/singing	Acitivity: all	0.034
	Body related	Object: body	–0.189
	Body related	Activity: physical (P) + movement (M)	−0.385*
Did the dream-plot appear…	Familiar/strange	Setting: unfamiliar + Character: unfamiliar	–0.264
	Pleasant/unpleasant	Negativity: Aggression + Failure + Misfortune	0.255
	Pleasant/unpleasant	Positivity: Friendliness + Success + Good Fortune	−0.379*

*Significant at the 0.05 level, **Significant at the 0.01 level.

A *post hoc* power analysis showed that the sample size of 30 dreams is sufficient (0.86) (Cohen, 1988).

## Discussion

This study provides a first validation of the DL-Q. While some of the DL-Q items were compared to the results of the HVDC, others were not covered by both instruments and a comparison was impossible. Therefore, only items of the second part of the questionnaire were included. Of those that were contrasted, dominant characters, the pleasantness of a dream, and body-related activity correlated significantly in both measures. These aspects might be those remembered most clearly and least affected by memory distortions. The findings suggest that the DL-Q is a promising way of analyzing dreams but this investigation does not allow general conclusions. Further validity and reliability studies should be done in the future to support the findings presented in this study. Unfortunately, an overall comparison to other dream questionnaires is difficult, since they do not include the same set of items or even the same overall research question. Furthermore, the DL-Q includes different types of response categories which enables participants to provide more adequate answers, however, statistical analysis is more complicated. Also, research with a bigger sample is needed in the future.

With the DL-Q, we hope to provide an easy-to-use tool for research and diagnostics. It facilitates the re-evaluation of the dream content in relation to the dreamer’s biography, goals and desires. The process of remembering, writing down, and evaluating a dream may be the initiator of a new look at things or might even lead to long-sought solutions for problems from the awake life. The mere act of writing down dreams regularly may enable to experience lucid dreams and take control of nightmares and issues that interfere with our daily functioning. The DL-Q can be applied as a complementary measure at home or in the sleep lab and can be filled in faster than other, more extensive instruments. In comparison with other dream questionnaires, the DL-Q is unique because it is actually a structured dream diary and can be used daily. This allows for longitudinal studies and the comparison of multiple dreams collected over a time period, e.g., during the course of psychological treatment. A short, and well-structured instrument such as this one may enable patients with cognitive impairments to better remember and structure their thoughts on the dreaming experience.

Unfortunately, dream questionnaires have some limitations. First, only a limited set of questions can be included, and important aspects of the dream might not be considered, or lead to a very vague description of the dream. Second, questions might be misunderstood by the dreamer, or produce answers based on one’s self-concept (Bernstein and Roberts, 1995). Third, the order and types of questions may also influence the dream content retrospectively. Moreover, the set of questions *per se* might help the dreamer to organize their thoughts and memories on one hand, but can lead to false, distorted memories on the other hand. To minimize such effects, the DL-Q includes the option of writing down a dream in one’s own words – which might compensate some of the disadvantages of questionnaires. One additional issue might be previously existing memory impairments, which we did not control for in this study. Other dream questionnaires, as mentioned above, are designed for certain subpopulations or research questions. The MADRE (Schredl et al., 2014) collects retrospective information about multiple dreams, how dreams were experienced on average over the last couple of months. Bernstein and Roberts (1995) developed a questionnaire (DCQ) based on the HCDC coding system, which asks about aspects typically experienced in dreams. They differ from the DL-Q, because they only ask for general characteristics of dreams and not about one dream in particular. The DL-Q might produce more accurate answers, built directly on one specific dream. Even though daily dream logs are generally considered to be more authentic and valid representatives of dream experiences, retrospective measures can be obtained with a single question, are less time-consuming than daily logs and more easily implemented in large-scale studies (Beaulieu-Prévost and Zadra, 2007). Therefore, motivation is higher for questionnaires and significantly more dreams are reported using questionnaires (Beaulieu-Prévost and Zadra, 2007). In conclusion, the DL-Q comprises both benefits of a dream diary and a questionnaire.

## Conclusion

Dreams are a significant part of the human existence and science has been striving to find a way of understanding dreams, as well as the subjective dreaming experience, for centuries. The validation of the DL-Q might open new opportunities for research and provide a fast and easy way to capture and re-evaluate the dreaming experience. The DL-Q is a flexible and open instrument that provides a number of basic items characterizing dreams which can be used separately or complementary to other methods, such as examinations in the sleep laboratory or dream diaries and other questionnaires evaluating psychological disorders, consciousness, coping styles or other specific topics. It might help us understand psychological and cognitive processes underlying the dream experience that are invisible and often not accessible for the dreamer until re-evaluating the dream content and its subjective meaning.

## Data Availability Statement

The raw data supporting the conclusions of this article will be made available by the authors, without undue reservation.

## Ethics Statement

Ethical review and approval was not required for the study on human participants in accordance with the local legislation and institutional requirements. The patients/participants provided their written informed consent to participate in this study.

## Author Contributions

BH and LM conducted the literature search, selected the eligible studies, conducted the statistical analysis and drafted the manuscript. IB and FN coded and evaluated the results of the HVDC. GK was part of the development of the DL-Q and involved in the statistical analysis. All authors confirm being the only contributors of this work and have approved it for publication.

## Conflict of Interest

The authors declare that the research was conducted in the absence of any commercial or financial relationships that could be construed as a potential conflict of interest.

## Supplementary Material

Supplementary Data Sheet 1 DREAMLAND II Questionnaire.
