# Supplementary material for: The Dreamland: Validation of a Structured Dream Diary
Source: Front Psychol. 2020 Oct 16;11:585702. doi: 10.3389/fpsyg.2020.585702 (PMC7596900; doi:10.3389/fpsyg.2020.585702)
Supplement: Supplementary Data Sheet 1 — DREAMLAND II Questionnaire. [file Data_Sheet_1.pdf]

DREAMLAND II

(Version 2.1. G. Klösch, B. Holzinger)

1. DID YOU DREAM LAST NIGHT?

□ No, I did not dream.

□ Yes, I dreamed, but I cannot remember anything anymore.

□ Yes, I dreamed, but I cannot remember details anymore.

□ Yes, I dreamed, and I still can remember details.

□ I am not sure if I dreamed.

2. HOW MANY DREAMS OF LAST NIGHT DO YOU REMEMBER?

□ One dream

□ Two dreams

□ More than two dreams

□ I don't know

PLEASE ANSWER TO FOLLOWING QUESTIONS ONLY IF YOU HAVE DREAMED SOMETHING OR IF YOU REMEMBER AT LEAST TO SOME DETAILS.

3. APPROXIMATELY HOW LONG DID YOUR DREAMS LAST?

□ Less than 5 minutes

□ 5 - 10 minutes

□ 11 - 30 minutes

□ More than 30 minutes

□ I don't know

4. DURING WHICH PART OF THE NIGHT WERE YOU DREAMING (MULTIPLE ANSWERS POSSIBLE)?

□ First part of the night

□ Second part of the night

□ Third part of the night

□ I don't know

5. HOW WOULD YOU DESCRIBE YOUR PERCEPTION OF TIME WHILE YOU WERE DREAMING.

□ … like in the waking state

□ … longer, slowed down

□ … shorter/faster

□ … I don't know

6. HOW MUCH DO YOU STILL REMEMBER OF YOUR DREAMS?

□ Everything, or nearly everything

□ Several details/episodes

□ Few details/episodes

□ Only some details

□ I cannot remember any details

7. DID YOU WAKE UP BECAUSE OF ONE OF YOUR DREAMS?

□ Yes

□ No

□ I don't know

7.1 IF SO, DID THIS DREAM MAKE IT HARDER FOR YOU TO GO BACK TO SLEEP?

□ Yes

□ No

□ I don't know

Please chose the most impressive dream of last night and write it down in as much detail as possible:

PLEASE CHARACTERISE ACCORDING TO THE FOLLOWING BULLETPOINTS THE DREAM OF LAST NIGHT WHICH YOU HAVE WRITTEN DOWN JUST NOW (MORE THAN ONE ANSWER POSSIBLE):

8. WHAT APPEARED IN YOUR DREAM?

□ Friends

□ Relatives

□ Colleagues/employer

□ Acquaintances

□ Strangers
□ Your own home
□ Familiar building
□ Known towns/ villages
□ Unknown towns/ villages
□ Indeterminable locations
□ Nature/landscapes
□ Known objects
□ Unknown/undefinable objects
□ Vehicles
□ Animals
□ Plants
□ Unknown/undefinable living beings
□ Others: ____________________

9. WAS THE CONTENT OF THE DREAM RELATED TO ….

□ … a known situation from everyday life
□ … a current event
□ … an event from recent past
□ … an event from the childhood
□ … a future event
□ … no known or event that can be remembered

10. DID THE DREAMPLOT APPEAR …

|―――――――――――――――――――――――|

strange familiar

|―――――――――――――――――――――――|

bizarre, not understandable logical, understandable

|―――――――――――――――――――――――|

colorful no color, black and white

If colorful, which was the predominant color in the dream? ___________

|―――――――――――――――――――――――|

pleasant unpleasant

|―――――――――――――――――――――――|

happy, funny serious, sad

|——————————————————————|

relaxing, enjoyable scary, threatening

|——————————————————————|

caring, gentle repulsing, aggressive

|——————————————————————|

sexual, compulsive asexual, not compulsive

|——————————————————————|

full of hope, optimistic despaired, pessimistic

11. PLEASE TRY TO CHARACTERISE THE PREDOMINANT TYPES OF SENSORY IMPRESSIONS:

Visual (optical impact)

|——————————————————————|

only visual not visual at all

Acoustic (related to hearing)

|——————————————————————|

Only acoustic not acoustic at all

Verbal expression (related to speech)

|——————————————————————|

Only verbal not verbal at all

Music/singing

|——————————————————————|

exclusively not at all

Odor/taste

|——————————————————————|

exclusively not at all

Tactile perception (for example perception of cold/warm, touch and so on)

|——————————————————————|

exclusively not at all

Body related events (for example: walking, running, jumping, flying and so on)

|————————————————————|

exclusively   not at all

12. HOW DID YOU PARTICIPATE IN THE DREAM?

□ actively participated in the dream plot

□ passively involved in the dream plot

□ observed the dream plot as a neutral observer

□ I don’t know

13. LUCID DREAMS

Lucid dreams are dreams in which we are fully aware of being in a dream and in which we can act according to our own decision.

13.1 While dreaming, were you aware to be dreaming?

□ Yes

□ No

□ I don’t know

13.2 Could you influence the dream according to your decisions?

□ Yes

□ No

□ I don’t know

14. WHEN DID YOU ANSWER TO THIS QUESTIONARY?

□ right after the dream (in the night or in the morning)

□ right after waking up

□ within the first hour after waking up

□ within the first six hours after waking up

□ later
